# Infant respiratory infections modulate lymphocyte homing to breast milk

**DOI:** 10.3389/fimmu.2024.1481416

**Published:** 2025-01-10

**Authors:** Yingying Zheng, Simone Corrêa-Silva, Regina Maria Rodrigues, Eloisa Corrêa de Souza, Fernanda A. Macaferri da Fonseca, Alfredo Elias Gilio, Magda Carneiro-Sampaio, Patricia Palmeira

**Affiliations:** ^1^ Department of Pediatrics, Faculdade de Medicina, Universidade de Sao Paulo, Sao Paulo, Brazil; ^2^ Laboratorio de Pediatria Clinica (LIM36), Hospital das Clinicas HCFMUSP, Faculdade de Medicina, Universidade de Sao Paulo, Sao Paulo, Brazil; ^3^ Department of Pediatrics, University Hospital, Medical School, University of São Paulo, São Paulo, Brazil

**Keywords:** breast milk, infant respiratory infections, mucosal immunology, lymphocyte homing, chemokine receptor

## Abstract

**Introduction:**

Chemokines and their receptors are essential for leukocyte migration to several tissues, including human milk. Here, we evaluated the homing of T and B lymphocyte subsets to breast milk in response to ongoing respiratory infections in the nursing infant.

**Methods:**

Blood and mature milk were collected from healthy mothers of nurslings with respiratory infections (Group I) and from healthy mothers of healthy nurslings (Group C). Total lymphocyte, T and B cells, their subset numbers, and the expression of the homing receptors CCR5, CCR6, CCR10, and CXCR3 in these cells were evaluated in milk. Maternal serum and milk chemokine, cytokine, and IgA and IgG antibody levels were also quantified.

**Results:**

All milk lymphocyte numbers were greater in Group I than in Group C. All CD4 T-cell subsets expressing CCR5, CCR6, and CXCR3 were higher in Group I. Within the CD8 T-cell subsets, only CCR6 and CXCR3 were higher in Group I, while CCR5 expression was higher in Group I exclusively for activated CD8 T cells. Group I showed greater numbers of all CCR6+ B-cell subsets and CXCR3+ naive B cells and plasma cells than did Group C. Infection of the nurslings promoted increased CCL20, CXCL10, IL-6, IL-8, total IgA, and IgG levels in the milk.

**Conclusion:**

Respiratory infections in nursing infants stimulate an increase in cytokines and chemokines in breast milk, facilitating the recruitment and activation of lymphocytes. This process may promote immunological tolerance and help in the maturation of the infant's immune system, providing an additional strategy for passive maternal-infant protection.

## 1 Introduction

It is commonly recognized that breast milk contains a high concentration of immune components that can protect newborns against a variety of infections and support the development of their own immune systems (1, 2). During the homeostatic state, the immunological composition of breast milk presents its highest level during the first 7 postpartum days (colostrum), after which it gradually decreases until it reaches a stable level, which is what we call mature milk (3).

Previous studies have revealed that the immunological composition of breast milk may depend on the maternal clinical status. For example, during mastitis, more leukocytes may be present in mature milk (4). Studies have also shown that infection in nursing infants can also promote an increase in maternal milk leukocytes. This is believed to occur because the pathogen contained in the infant’s saliva can be transferred to the mother’s breast during breastfeeding and thus can induce a local immune response in the mammary gland mucosa. An increase in leukocyte infiltration into the breast, triggered by inflammation, leads to leakage of these cells into milk (5, 6).

During respiratory infection, viruses are recognized by pattern recognition receptors (PRRs) present in mucosa epithelial cells, which results in the activation of transcription factors such as NF-κB and c-Jun and different IFN regulatory factors (IRFs). Activation of these transcription factors induces high production of proinflammatory cytokines, i.e., type I IFN, IL-6, TNF-α, and thymic stromal lymphopoietin (TSLP), and chemokines such as CCL5, CCL2, CXCL8 and CXCL10 (7). These factors are produced by dendritic cells (DCs) and alveolar macrophages in the respiratory tract (8) and are involved in the trafficking of several leukocyte types, such as monocytes, neutrophils, DCs, T cells, eosinophils, and NK cells, to inflammatory sites (9). The homing of leukocytes to different tissues, including mucosal sites such as the breast, is mediated by specific combinations of chemokine receptors and adhesion molecules. A previous study reported that the lactating breast compartment is more closely associated with the gut mucosa than with the upper respiratory tract mucosa (10). For instance, the chemokine receptor CCR10 is expressed on all plasma cells within mucosal compartments, while its ligand, CCL28, abundantly expressed in human milk and produced by most mucosal epithelial cells, specifically attracts IgA+ plasma cells to the colon lamina propria and secretory organs such as the salivary and mammary glands (11).

Children infected with respiratory syncytial virus exhibit elevated serum levels of CCL5 and CXCL10, which are involved in the homing of CCR5+ and CXCR3+ cells, respectively. In contrast, in adults, CCL5, CCL20, and especially CXCL9, CXCL10, and CXCL11 are markedly elevated (12), attracting CCR5+, CCR6+, and CXCR3+ cells. Furthermore, the CCL5/CCR5, CCL20/CCR6, and CXCL10/CXCR3 axes are also involved in the homing of immune cells to the intestine (13, 14).

Respiratory infections are the leading cause of morbidity and mortality in children younger than 5 years (15), and classical epidemiologic studies have reported the protective effects of breastfeeding against both acute and chronic otitis media (16, 17), as well as pneumonia (18, 19). It has been shown that formula-fed infants are three times more likely to be hospitalized due to severe respiratory diseases than breastfed infants (20). Additionally, total and specific IgA and IgG antibodies against several pathogens, such as SARS-CoV-2, pneumococcus, *Neisseria meningitidis*, influenza A, and pertussis toxin, can be induced in human milk after maternal natural infection or immunization, and these antibodies are able to neutralize pathogens and protect the nursing infants (2, 21–24).

T and B lymphocytes play important roles in the resolution of viral and bacterial infections, and the production of chemokines and the expression of the corresponding receptors in these cells may lead to successful homing to inflammatory sites. An increase in the general population of T and B lymphocytes and macrophages in human milk has already been demonstrated following infant infection (4–6), but the subtypes of these lymphocytes and the chemotactic substances and adherence molecules involved in the homing of these cells to the mammary mucosal tissue and, consequently, into the milk, have not yet been described (25). A previous study by our group revealed significant changes in macrophage subtypes, as well as in the levels of chemokines and their specific receptors present in these breast milk cells, in nursing infants with respiratory infections from healthy mothers (6).

The change in the influx of breast milk leukocytes during current respiratory infections in infants is likely another biological mechanism by which breast milk protects the nursing infant. Here, we analyzed T and B lymphocyte subsets, the production of chemokines, and the expression of the corresponding receptors involved in the homing of these cells to breast milk during ongoing respiratory infections in nursing infants.

## 2 Materials and methods

This prospective cross-sectional study was approved by the Research Ethics Committee of the participating institutions (CAPPesq protocol 1.754.872 and CEP-HU/USP protocol 1.786.170) and was carried out between March 2017 and June 2019. All guidelines for human experimentation were followed, and no samples were collected from the nursing infants. After providing informed consent, maternal peripheral blood and mature breast milk samples were collected in the Emergency Room for Children and the Pediatric Ward of the University Hospital of Universidade de São Paulo and from Dr. Regina Maria Rodrigues’ Private Pediatric Clinic. Two groups of healthy mothers were designated: Group I, composed of 31 healthy mothers of nursing infants with acute respiratory infections, and the control group, composed of 24 healthy mothers of healthy nursing infants (Group C). The inclusion criteria were as follows: healthy nursing women older than 20 years of age whose infants were born at full term with adequate weight for gestational age and who were between 1 and 11 months of age at the time of collection. For Group C, the infants were healthy at the time of maternal blood and milk collection. For Group I, the infants entered the emergency room service with respiratory infections, and the disease was confirmed through clinical symptoms by the medical board and/or by laboratory results when available (**Table 1**). The exclusion criteria included the following maternal characteristics: chronic diseases; positive serology for conventional serologic tests, use of anti-inflammatory, immunosuppressive, or immunomodulatory medications; or the presence of mastitis, fever, flu or cold, cough, pneumonia, diarrhea, vomiting, or allergies during the collection period. For the healthy infant group, no disease symptoms were noted in the nursing infant for 7 days before sample collection. Maternal blood was collected from a peripheral vein in polymer gel tubes for serum separation, after which the serum was aliquoted and stored at −80°C. Breast milk samples were collected by manual expression and immediately transported to the laboratory under refrigeration. All volumes were recorded to calculate the number of cells per mL (numbers/mL).

**Table 1:** Demographics from the mothers and nursing infants of Group I (infected) and Group C (healthy).

Parameters	Group I	Group C	p
	(n = 31)	(n = 24)	
Mothers' Age	27 ± 1	32 ± 1	***0.011***
Parity, n *	2 ± 1	1 ± 1	***0.0167***
Gestational age at delivery (weeks) ^#^	39 (38 – 39)	39 (38 – 39)	
Mode of delivery			
Cesarean Section, n (%)	12 (39%)	17 (70%)	***0.0215***
Vaginal delivery, n (%)	19 (61%)	7 (30%)	
Infants' Age	4 (3 – 5)	4 (4 - 6)	*0.2668*
Male, n (%)	19 (61%)	8 (33%)	*0.1173*
Breastfeeding type, n (%):* *			
Exclusive^1^	18 (58%)	16 (66%)	*0.9438*
Predominant^2^	0	0	* *
Complementary^3^	13 (42%)	8 (34%)	
Breastfeeding frequency:* *			
- On demand	28 (91%)	22 (92%)	*0.6056*
**Symptom onset (days)** ^#^	7 (5 - 9)	NA	
Symptom			
- Cough	31 (100%)	NA	
- Rhinorrhea	28 (91%)		
- Fever	23 (75%)		
- Vomit	0		
Diagnosis			
Acute bronchiolitis, total, n (%)	26 (84%)	NA	
- VSR, only	10 (32%)		
- VSR and otitis media	2 (6.5%)		
- Parainfluenza III, only	2 (6.5%)		
- Non-specific	12 (39%)		
Pneumonia, total, n (%)	5 (16%)		
- VSR and *Klebsiella pneumoniae*	1 (3%)		
- Non-specific	3 (10%)		
- Non-specific and otitis media	1 (3%)		

*Results expressed as means (± standard deviation) were analyzed by Student’s t test; ^#^Results expressed as medians (upper and lower CI 95%) analyzed by the Mann-Whitney; ^1^Exclusive breastfeeding; ^2^Receives some types of liquids (water and water-based drinks, fruit juices); ^3^Receives any type of food or liquids including non-human milk or formula; RSV: respiratory syncytial virus; NA: Not applicable. The significant p-values are in bold.

### 2.1 Immunophenotyping by flow cytometry

Breast milk samples were defatted by centrifugation, and the milk serum and cell pellet were collected. After two washes with BD FACSFlow™ (BD Biosciences, San Jose, CA, USA), the cells were resuspended in 1 mL of RPMI medium supplemented with 10% fetal calf serum, and the total leukocyte numbers in the breast milk were determined with a Neubauer chamber. The viability of human milk cells for all samples was assessed using Trypan Blue staining, with the proportion of non-viable cells determined by comparing the number of stained (non-viable) cells to the total cell count, yielding approximately 95% viable cells. A concentration of 1.6 × 10^6^ cells/mL was used for immunophenotyping analysis on the same day, with all samples included in each experiment. The milk serum was immediately stored at −80°C for cytokine and chemokine measurements.

The cells were stained with fluorochrome-conjugated monoclonal antibodies against each cell population under study for 30 minutes. After two washes, the cells were resuspended in BD FACSFlow™ and immediately analyzed to prevent the loss of fluorescence. A total of 100,000 events were acquired with a BD LSRII Flow Cytometer™ (BD Biosciences) with FACSDiva software (Becton Dickinson), and the analysis was performed using FlowJo software (Tree Star, Ashland, OR, USA).

Milk leukocytes were placed in a CD45+ P1 gate based on forward- and side-scatter characteristics (FSC-A X SSC-A). The CD4+ and CD8+ T-cell compartments were characterized using combinations of markers gating on CD3+/CD4+ or CD3+/CD8+ cells within the total lymphocyte population. Within the CD4+ or CD8+ T lymphocyte gates, CD45RA+ cells were naive T lymphocytes, CD45RA- cells were memory T lymphocytes, and CD69+ cells were activated T lymphocytes. B cells were identified as CD19+ cells within the total lymphocyte population. Definitions of B-cell subsets were according to the following markers: CD19+/CD27- were naive B lymphocytes; CD19+/CD27+ cells were memory B lymphocytes; and CD27+CD38+CD138+ were plasma cells. The chemokine receptors CCR5, CCR6, CCR10, and CXCR3 were analyzed in all T- and B-cell subpopulations. The data are presented as absolute numbers, which were calculated from the complete leukocyte counts. The median fluorescence intensity (MFI) of chemokine receptors within the T- and B-cell subsets was determined for all the milk samples.

### 2.2 Serum and milk chemokines and cytokines

The serum and milk chemokines CCL5, CCL20, CCL28, and CXCL10 were measured by ELISA (R&D Systems, USA). IL-1β, IL-6, IL-8, IL-10, IL-12, and TNF-α were measured by flow cytometry using Cytometric Bead Array (CBA, BD Biosciences, San Jose, CA, USA). All these assays were performed according to the manufacturer’s instructions.

### 2.3 Milk total antibodies

Total IgA antibodies in the breast milk were measured by ELISA as previously described by Nagao et al. (26) with modifications.

Total IgG antibodies in milk were determined by ELISA following our previously standardized protocol as follows: an aliquot of 10 µg/mL purified anti-IgG antibody suspension diluted in PBS 7.4 (I-1136, Sigma, St. Louis, MO, USA) was used for coating the microplates (Costar, Cambridge, MA, USA), which were maintained for 16 to 18 hours at 4°C. After blocking with 10% nonfat milk diluted in PBS at room temperature, the samples were incubated in duplicate in four serial dilution steps diluted in PBS-0.5 M NaCl-0.2% Tween 20 with 1% nonfat milk for 2 hours at 37°C. Then, the plates were incubated with 2.5 µg/mL peroxidase-conjugated anti-human IgG (HP6043-HRP, Hybridoma, Baltimore, MD, USA) for 2 hours at 37°C, and a substrate solution containing 0.4 mg ortophenylenediamine/ml in 0.1 M citrate-phosphate buffer, pH 5.0, was added to the plates. After a 30 min incubation, the reaction was stopped with 50 µL of 2.5 N H_2_SO_4_. The absorbances were read in a microplate reader at 492 nm. The plates were washed with PBS–0.1% Tween between each step. Purified human IgG (I-2511, Sigma, St. Louis, MO, USA) was used as the primary reference standard at concentrations ranging from 250 to 3.9 ng/mL, and the total IgG concentrations in the milk samples were expressed as µg/mL.

### 2.4 Statistical analysis

The statistical and graphical analyses were performed in GraphPad Prism version 7.0 (GraphPad Software, La Jolla, CA, USA). In addition to descriptive analyses, which were used to calculate the means, medians, standard deviations, and confidence intervals, the normality of the data was performed by the D’Agostino-Pearson normality test. Mann−Whitney or unpaired Student’s t tests were used to examine differences between groups, depending upon the normality of the data. Comparisons of chemokine receptor expression were performed using the Kruskal–Wallis test, followed by Dunn’s multiple comparisons *post hoc* test. The Wilcoxon signed rank test or paired Student’s t test was used to analyze the differences between blood and milk within the same group. Spearman’s correlation analysis was performed to verify the association between chemokines and cytokines, and to analyze the association between maternal parity and age with antibodies, cytokines/chemokines and leukocytes.

A confidence limit of 95% and a significance threshold of 0.05 were applied for all the statistical tests.

## 3 Results

### 3.1 Maternal and infant demographics and clinical data

More than half of the nursing infants in both groups were exclusively breastfed, and almost all were breastfed on demand, indicating that breast stimulation was constant.

All the infants in Group I were diagnosed with respiratory infections: 26 with acute bronchiolitis, and 5 with pneumonia. The onset of symptoms occurred between 3 and 7 days before the milk collection. Among the sick children, 64% presented significant changes in chest radiography, such as hyperinflation, atelectasis, or infiltration. All the infants presented with cough, 91% had rhinorrhea, and 75% had fever. All infants received medical support, and the treatment consisted mainly of inhalation of saline, with or without bronchodilators and, when necessary, cortisol, antibiotics, or antipyretic agents, according to the infants’ symptoms and diagnosis.

The maternal and infant demographics, and breastfeeding frequency of the two groups, and diagnosis and symptoms from the infants of Group I are summarized in **Table 1**.

### 3.2 Numbers of breast milk T and B lymphocytes

There were more total leukocytes in the breast milk of Group I than in the control group (median: Group I: 5.5x10^4^/mL and Group C: 2.7x10^4^/mL, *p=0.0286*). In agreement, **Table 2** shows that the total numbers of CD3+, CD4+, and CD8+ T cells and all their subsets (naïve, activated, and memory cells) in the milk of the healthy mothers of the infected nurslings were greater than those in milk from the healthy mothers of healthy infants. **Table 2** also shows the numbers of total B lymphocytes and their subsets: naive, memory, and plasma cells, which were consistently greater in the breast milk from Group I than in that from Group C.

**Table 2:** Absolute numbers (/mL) of milk total, naive, memory and activated CD4+ and CD8+ T cells and the total, naive, and memory B lymphocytes and plasma cells from mothers of infected (Group I) and healthy (Group C) nursing infants.

	Milk		*p*
	Infected	Control	
**Total CD3**	10,613 (5,071-52,246)	1,180 (1,031-2,984)	***p < 0.0001***
**Total CD4**	6,227 (2,801-34,790)	723 (541-1,770)	***p < 0.0001***
**Naive CD45RA+**	2,791 (2,326-18,612)	437 (302-758)	***p < 0.0001***
**Memory CD45RA-**	2,971 (0-16,937)	214 (61-1,189)	***p = 0.0004***
**Activated CD69+**	3,605 (297-31,289)	487 (219-1,466)	***p < 0.0001***
**Total CD8**	3,672 (1,940-18,381)	653 (318-1,386)	***p = 0.0060***
**Naive CD45RA+**	1,524 (917-11,447)	263 (101-844)	***p = 0.0121***
**Memory CD45RA-**	1,131 (907-7,050)	155 (150-609)	***p = 0.003*5**
**Activated CD69+**	1,387 (357-13,087)	142 (29-538)	***p = 0.0002***
**Total CD19+**	6,656 (2,247-36,468)	1,295 (339-4,595)	***p = 0.0005***
**Naive CD27-**	1,083 (516-13,505)	218 (0-1,977)	***p = 0.0019***
**Memory CD27+**	3,828 (1,629-23,055)	868 (560-2,676)	***p = 0.000*7**
**Plasma cell**	1,594 (1,298-4,528)	659 (475-1,855)	*p = 0.0531*

Results expressed as means (± standard deviation) were analyzed by Student’s t test; Results expressed as medians (upper and lower CI 95%) analyzed by the Mann-Whitney Test. The significant p-values are in bold.

### 3.3 Expression of chemokine receptors in T lymphocyte subsets from breast milk

**Figure 1:**
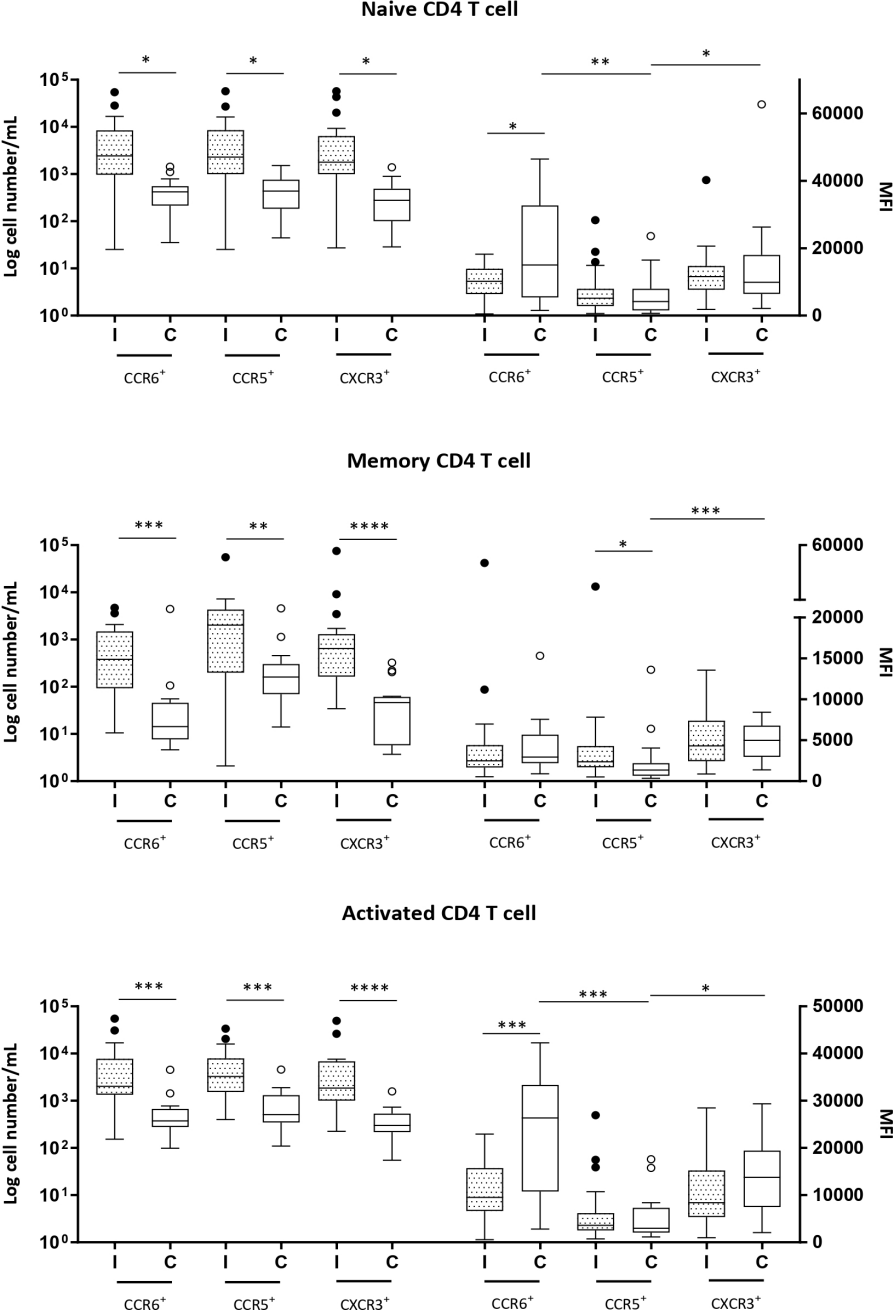
Naive (CD3^+^CD4^+^CD45RA^+^), memory (CD3^+^CD4^+^CD45RA^−^) and activated (CD3^+^CD4^+^CD69^+^) CD4^+^ T cells expressing CCR6, CCR5 and CXCR3 in milk samples from mothers of infected (Group l) and healthy (Group C) nurslings. Log cell numbers/mL (left y-axis) and median fluorescence intensity (MFI) (right y-axis). Box plots: black horizontal lines are medians, the solid lines of the box represent the 75th and 25th percentiles, and the short lines outside the top and the base of the box represent the highest and the lowest values, respectively. *p < 0.05; **p < 0.01; ***p < 0.001; ****p < 0.0001.

**Figure 2:**
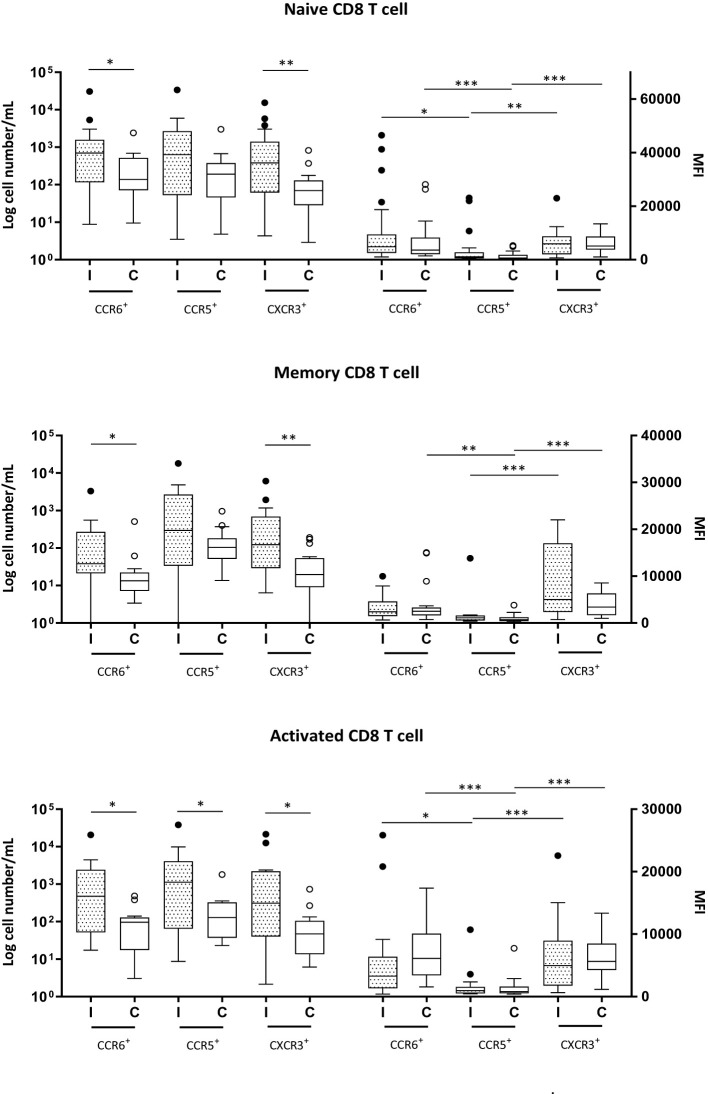
Naive (CD3^+^CD8^+^CD45RA^+^), memory (CD3^+^CD8^+^CD45RA^+^) and activated (CD3^+^CD8^+^CD69^+^) CD8^+^ T cells expressing CCR6, CCR5 and CXCR3 in milk samples from mothers of infected (Group I) and healthy (Group C) nurslings. Log cell numbers/mL (left y-axis) and median fluorescence intensity (MFI) (right y-axis). Box plots: black horizontal lines are medians, the solid lines of the box represent the 75th and 25th percentiles, and the short lines outside the top and the base of the box represent the highest and the lowest values, respectively. *p < 0.05; **p < 0.01; ***p < 0.001.

The numbers of naive, memory, and activated CD4+ and CD8+ T cells expressing chemokine receptors were markedly increased in Group I, except for CCR5+ naive and memory CD8+ T cells, which showed similar numbers as those in the control group (**Figures 1**, **2**).

MFI analysis revealed that in naive and activated CD4+ T cells in Group C, the expression of CCR5 was lower than that of CCR6 or CXCR3. However, during infant infection, this difference was lost, and the expressions of CCR6, CCR5, and CXCR3 were found to be equivalent. This is because CCR6 expression decreased during infant infection (**Figure 1**). CCR5 expression was lower than CXCR3 expression in the memory CD4+ T cells of the control group (Group C). However, during infection, CCR5 expression increased and the difference between the expression of the receptors was lost (**Figure 1**).

In all CD8+ T-cell subsets, the expression of chemokine receptors was similar between Groups I and C. CCR6 and CXCR3 were expressed at higher levels than CCR5 in naive and activated CD8+ T cells, regardless of the health condition of the infant (**Figure 2**). In memory CD8+ T cells, during infection, only CXCR3 presented greater expression than CCR5, while in the control group, both CXCR3 and CCR6 exhibited greater expression than CCR5 (**Figure 2**).

### 3.4 Expression of chemokine receptors in B lymphocyte subsets from breast milk

**Figure 3:**
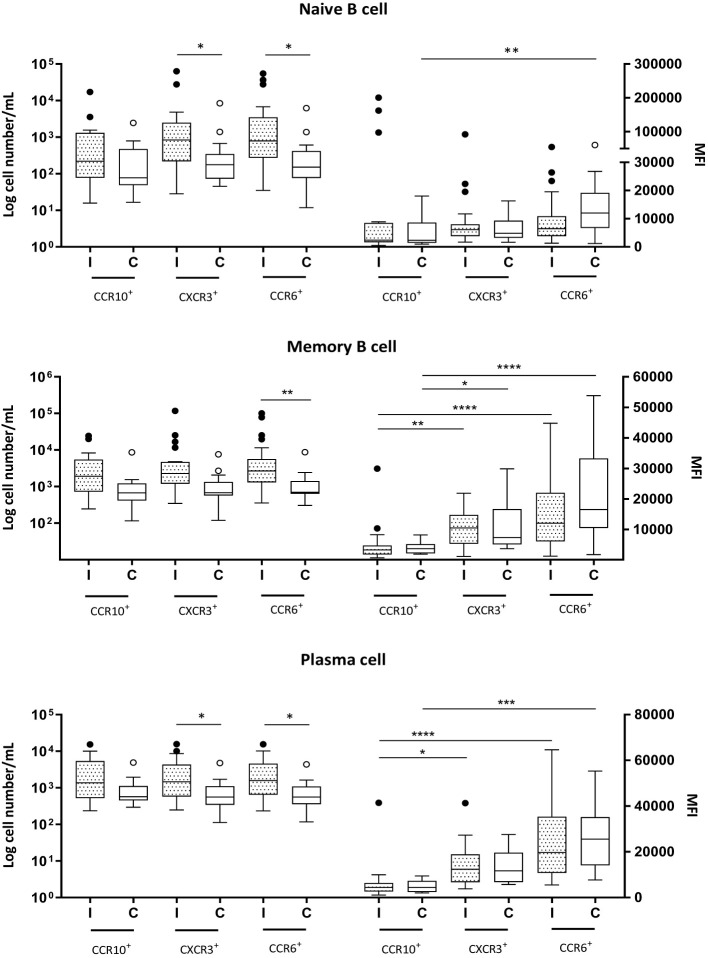
Naive (CD19^+^CD27^−^) and memory (CD19^+^CD27^+^) B cells and plasma cells (CD19^+^CD27^+^CD38^+^CD138^+^) expressing CCR10, CXCR3 and CCR6 in milk samples from mothers of infected (Group l) and healthy (Group C) nursing infants. Log cell numbers/mL (left y-axis) and median fluorescence intensity (MFI) (right y-axis). Box plots: black horizontal lines are medians, the solid lines of the box represent the 75th and 25th percentiles, and the short lines outside the top and the base of the box represent the highest and the lowest values, respectively. *p < 0.05; **p < 0.01; ***p < 0.001; ****p < 0.0001.

Group I exhibited greater numbers of all CCR6^+^ B-cell subsets, as well as CXCR3^+^ naive and plasma cell subsets, than did Group C (**Figure 3**).

The analyses also revealed no differences in receptor expression between Groups I and C for any of the B-cell subpopulations. In naive cells, the expression levels of CCR10, CXCR3, and CCR6 were similar during infection. However, in Group C, the expression of CCR6 was greater than that of CCR10 (**Figure 3**). The MFIs of the receptors CCR10, CXCR3, and CCR6 were found to be closely similar in memory B cells and plasma cells across both groups; CCR10 had the lowest expression, and CCR6 had the highest expression, regardless of the infant’s health condition (**Figure 3**).

### 3.5 Serum and breast milk chemokines and cytokines

All chemokines CCL20, CCL28, and CXCL10 levels were greater in breast milk than in maternal serum, except for CCL5, which was markedly lower in breast milk than in the serum, regardless of the infant’s health status. In the milk, during infant infection, CCL20 and CXCL10 concentrations were greater in Group I than in Group C, and CXCL10 was the only chemokine that increased in the mother’s serum during infant infection. CCL28 presented a greater concentration in the milk than did the other evaluated chemokines; however, it was the only chemokine whose concentration remained unchanged in milk during infant infection (**Table 3**).

**Table 3:** CCL5, CCL20, CCL28 and CXCL10 chemokine levels and IL-6 and IL-8 levels in the serum and milk from mothers of infected and healthy (control) nursing infants.

pg/mL	Serum			Milk			Infected	Control
	Infected	Control	*p*	Infected	Control	*p*	B X M *(p)*	B X M *(p)*
**CCL5**	21,748 (22,196-34,310)	25,299 (22,494-34,420)	*0.7169*	17.5 (3.7-31.3)	8.1 (7.6-8.6)	***0.0451***	***<0*.*0001***	***<0*.*0001***
**CCL20**	78 (42.2-80.6)	78 (45.3-88.4)	*0.9965*	329.9 ± 58.2	152.1 ± 24.0	***0.0128***	***<0*.*0001***	***0.0083***
**CCL28**	62.5 (283.4-1,704)	66.2 (106.9-898)	*0.7888*	91,676 (81,726-117,857)	112,935 (103,282-156,392)	*0.0759*	***<0*.*0001***	***<0*.*0001***
**CXCL10**	25.7 (22.8-49)	15.6 (0-91.2)	***0.0346***	6,650 ± 895	2,025 ± 404	***0.0003***	***<0*.*0001***	***<0*.*0001***
**IL-6**	1.4 (1.2-2.2)	1.1 (0.5-2.9)	*0.1449*	4.8 (4.4-19.7)	1.7 (0.2-12.6)	***0.0021***	***<0*.*0001***	*0.1594*
**IL-8**	5.5 (5.4-9.9)	7. 9 (6.3-10.6)	*0.0828*	319 (449.1-981.6)	193.2 (169.9-566.5)	***0.0103***	***<0*.*0001***	***<0*.*0001***

B: Blood, M: Milk. Results expressed as means (± standard deviation) were analyzed by Student’s t test; Results expressed as medians (upper and lower CI 95%) analyzed by the Mann-Whitney Test. The significant p-values are in bold.

The IL-6 and IL-8 concentrations in the milk were greater in Group I than in Group C, while in the serum, no differences were detected between the groups. Human milk IL-8 levels were always greater than those in serum, but the milk IL-6 concentrations were greater than those in the serum only when the nurslings were infected (**Table 3**). No differences were found in the IL-1β, IL-10, IL-12 and TNF-α levels between the groups, but IL-1β showed higher levels in milk compared to maternal serum exclusively in Group I (**Supplementary Table 1**).

### 3.6 Correlation analyses

Spearman’s correlation analyses were performed to verify the possible influence of cytokines on chemokine concentrations in the milk of each group (**Table 4**). In the milk, almost all chemokines were positively correlated with IL-6 and IL-8 levels in Group I, while in Group C, only the IL-6 level was positively correlated with CCL5. Additionally, in the milk, CCL28 levels were correlated negatively with IL-8 levels in Group I, but not in Group C.

**Table 4:** Correlation indices between chemokines and cytokines in milk from mothers of infected and healthy nursing infants.

Milk				
	Infected		Control	
	IL-6	IL-8	IL-6	IL-8
**CCL5**	0.46^*^	0.52^**^	0.41^*^	–
**CCL20**	0.61^***^	0.49^**^	–	–
**CCL28**	–	- 0.41^*^	–	–
**CXCL10**	0.73^****^	0.65^***^	–	–

^*^p <0.05; ^**^p< 0.01; ^***^p<0.001; ^****^p<0.0001.

The Spearman correlation analysis performed to verify the association between the maternal age and parity with antibodies, cytokines/chemokines and leukocytes is shown in **Supplementary Table 2**. The correlation analysis did not reveal association between those parameters and maternal age or parity, with the exception of a weak, but significant positive correlation between parity and IL-8 (r= 0.382, *p=0.03*) and a negative correlation between parity and plasma cells (r= -0.508, *p=0.04*).

### 3.7 IgA and IgG concentrations in breast milk

**Figure 4:**
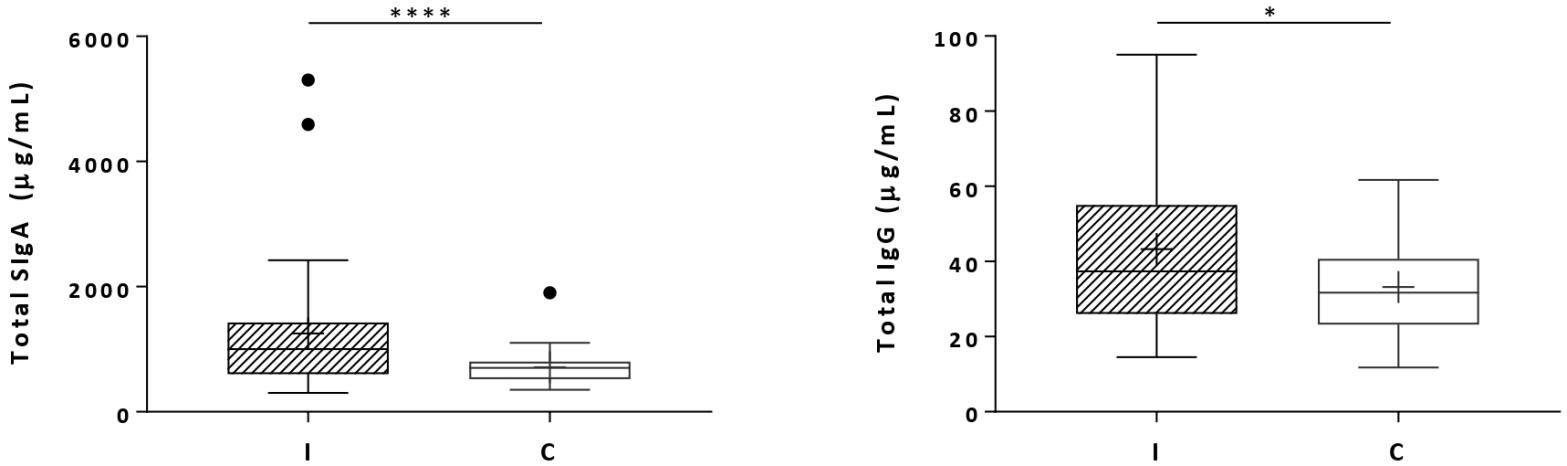
Total secretory IgA (SigA) and total IgG concentrations in milk samples from mothers of infected (Group I) and healthy (Group C) nursing infants. Box plots: black crosses are the mean, black horizontal lines are medians, the solid lines of the box represent the 75th and 25th percentiles, and the short lines outside the top and the base of the box represent the highest and the lowest values, respectively. *p < 0.05; ****p < 0.0001.

Total secretory IgA and total IgG concentrations were increased in the milk during infant infection (**Figure 4**). The results showed that the IgA concentrations were 25 times greater than the IgG concentrations, and the difference between the groups was more pronounced.

## 4 Discussion

In the present study, we addressed how ongoing respiratory infections in nursing infants affect T and B lymphocyte profiles and their migration to breast milk by analyzing several chemokines and their receptors.

Group I was composed of mothers of infants with respiratory infections that are mostly caused by viruses, such as respiratory syncytial viruses A and B (RSV), rhinoviruses, parainfluenza viruses 1–4, influenza viruses A-B, adenoviruses, human metapneumovirus (HMPV), human bocavirus (HBoV) and coronavirus (SARS-CoV-2 was not included in the present study because sample collection was performed before the pandemic) (27). These viruses are present in nursing infant saliva and can be transferred to the breast during breastfeeding, where they can induce a local immune response that promotes the migration of several types of leukocytes due to the increase in the concentrations of several chemokines in the mammary gland.

In the present study, we observed an increase in milk IP-10/CXCL10, which promoted an increase in CXCR3+ CD4+ and CD8+ T lymphocytes and B lymphocytes in milk. This is expected because, as mentioned above, the nursing infants in Group I manifested predominantly respiratory infections and pneumonia, and CXCL10, as well as the CXCR3 receptor, is highly induced by IFN-γ and type I IFN via NF-κB activation during infection, injury, or inflammatory responses, such as multiple sclerosis, skin and mucous membrane inflammation and bronchiolitis (28–34).

An increase in CCL5 levels in milk is expected because this chemokine is produced early during virus infection and can also be induced later in response to TNF-α and IFN-γ produced by CD4+ and CD8+ T cells, epithelial cells, fibroblasts, and platelets (35–37). However, no differences were observed between Group I and Group C. Furthermore, CCL5 levels in breast milk are much lower than those in serum, raising doubts about its significance for leukocyte recruitment compared to other chemokines found in high quantities in milk. On the other hand, although CCR5 expression (MFI) was low in all T lymphocyte subsets, suggesting that CCR5 is not the main chemokine receptor involved in the trafficking of T lymphocytes into milk, we observed increased numbers of all CCR5+CD4+ T-cell subsets and activated CCR5+CD8+ T cells in breast milk from mothers of infected infants, even with an equivalent MFI. Although CCR5 is one of the corresponding receptors for CCL5, it can also be chemoattracted by other chemokines, including CCL3 and CCL4, whose levels were not analyzed in this study.

The production of CCR5 and CXCR3 ligands is rapidly induced after a secondary challenge with a respiratory virus, CXCR3 is highly expressed on virus-specific cells, and the prompt recruitment of CD8+ memory T cells to the pulmonary airways is entirely dependent on the rapid expression of CCR5 by these cells (38). CCR5 and CXCR3 are expressed in human lamina propria lymphocytes (LPLs) and intraepithelial lymphocytes (IELs) and play important roles in recruiting leukocytes to the gut in patients with inflammatory bowel disease (13). Under homeostatic conditions, the colonic epithelium expresses low levels of CXCL9, CXCL10, and CXCL11. However, under inflammatory conditions, such as colitis and Crohn’s disease, and infections, like COVID-19, the production of mainly CXCL10 strongly increases, which is associated with high tissue infiltration of activated CXCR3+ T cells (39–43). Milk from mothers of infected infants exhibited a similar response, with increased CXCL10 levels leading to a significant rise, particularly in CXCR3+ activated and memory CD4+ T cells.

Here, we observed that respiratory infections in nursing infants also induced higher CCL20 concentrations in breast milk, leading to an increase in all CCR6+ T- and B-cell subpopulations. CCL20 is typically present at low levels, as we showed in the maternal serum from both groups, but was increased in the breast milk from the mothers in Group I. CCL20 is constitutively expressed by isolated lymphoid follicles and follicle-associated epithelium overlying Peyer’s patches, and it acts as both an inflammatory and homeostatic chemokine, contributing to homeostasis and maintenance of the mucosa (14). Previous studies have demonstrated that CCL20 can be strongly induced by proinflammatory cytokines and TLR agonists and is notably increased in mucosal inflammatory diseases, as is the expression of CCR6 in specific CD4+ T-cell subpopulations (44, 45). CCR6 is present in most B cells, and its ligand, CCL20, plays an important role in mucosal homeostasis, attracting not only B cells but also immature DC populations and memory T cells under normal conditions, as these subpopulations preferentially target these sites of continuous antigenic challenge (44).

Our results have shown that ongoing respiratory infections in nursing infants promoted an increase in the concentrations of milk chemokines (CCL20 and CXCL10), probably induced by the release of cytokines (IL-6 and IL-8) into the breast, as shown by the positive correlation indices, resulting in increased numbers of T and B cells expressing the corresponding chemokine receptors (CCR6 and CXCR3, respectively) in milk. CCL28 is highly produced by exocrine glands, including the mammary glands (46), which explains why, in the present study, CCL28 was the most prevalent chemokine in breast milk. However, the levels of CCL28 in the milk from Group I did not show a significant change, which may explain why there was no difference in all CCR10+ B-cell subset numbers between the I and C groups. These results were further reinforced by the low CCR10 expression (MFI) in memory B-cell and plasma cell subsets. Since the main function of these cells is the maintenance of homeostasis, a higher migration of CCR10+ B cells into milk may not be as relevant during infection (47, 48).

On the other hand, the increase in CXCR3+ plasma cell numbers in the milk from the mothers in Group I indicates the trafficking of these cells, possibly IgG+ plasma cells, to the breast mucosa and extravasation into milk. CXCL10 quantification in the blood and milk samples corroborated these results. Most B cells in breast milk exhibit an IgD- memory B-cell phenotype with a particular profile of adhesion molecules (CD44+, CD62L-, α4β7+/-, α4β1+), suggesting that these cells may originate from gut-associated lymphoid tissue (GALT) (10). Most of these cells are activated, and it has been shown that plasma cells present in breast milk produce mainly IgG antibodies (10). These authors suggest that the extravasation of B cells from the mammary epithelium to the milk may imply a negative selection process operating in the alveolar epithelium or the mammary duct. IgA+ memory B cells are thought to be more strongly retained in mammary mucosal tissue because of their high CCR10 expression and the abundant presence of CCL28, resulting in the accumulation of these cells and the production of high concentrations of IgA antibodies that flow into the milk (49). In contrast, IgG+ cells have a weaker attraction in response to CCL28, allowing those cells to migrate into the milk (50). Despite this, infant infection significantly stimulated the production of both IgA and IgG, in which the milk samples from Group I showed higher levels of these immunoglobulins than those from the control group.

Indeed, leukocyte subsets differ between the mammary gland and milk, suggesting the presence of selective mechanisms that regulate cell transmigration into milk. CD8+ T cells, along with CD4+ T cells, γδ T cells, and NK cells, may selectively translocate through the tight junctions of the mammary epithelium while maintaining its structural integrity (51), due to high expression of the genes (Cldn3, Cldn7, Tjp1) involved in the biosynthesis of the tight junction (TJ) protein ZO-1 and the regulation of claudin polymerization (52).

Higher production of chemokines is related to higher expression of IL-1β (53). However, our results have shown that in the milk and, probably, in the breast, the production of the chemokines CCL5, CXCL10, and CCL20 is strongly correlated with IL-6 and, to a lesser extent, with IL-8 (CXCL8), the latter only in Group I, but not with IL-1β or TNF-α, as described in the literature. Most chemokines, such as IL-8 (CXCL8), CCL20, CXCL9, CXCL10, CXCL11, and CX3CL1, exhibit an expression pattern that points to an important role in inflammation, and their production is induced by proinflammatory agents, particularly TNF-α, IL-1α, IL-1β and IFN-γ, or by bacterial LPS (54–56). Furthermore, prolactin stimulates the production of IL-8 by human mammary epithelial cells in culture, which may explain the higher levels of IL-8 observed in our milk samples than in the blood from both groups. We and others have detected IL-8 in human milk at concentrations high enough to explain the recruitment of leukocytes (57). High concentrations of IL-8 and CCL5 in breast milk can bind to proteoglycans in enterocytes (58), inducing adherence and diapedesis of maternal leukocytes in the infant’s intestinal tissue (59).

The inverse correlation of IL-8 with CCL28 in the milk from the mothers of infected infants shows the role of IL-8 (and IL-6) in the breast in response to the inflammatory stimulus provided by the infant, and its increase induces a decrease in the production of CCL28, which, in the breast, is fundamentally homeostatic and is a chemoattractant for IgA-producing plasma cells. However, in the control group, the absence of antigenic stimulation did not promote increased IL-8 or IL-6 production, and CCL28 remained high, suggesting that the mammary mucosal tissue of the mothers from the control group was in homeostasis.

Surprisingly, during infant infection, a positive correlation of IL-8 and a negative correlation of plasma cells with parity were observed. Few studies have examined the link between parity and immune components in human milk. Higher levels of IgA, IgM, IgG, and other immune factors were reported in the colostrum of primiparous mothers compared to multiparous mothers (60, 61). Results vary across lactation stages, as it was found that parity influenced IgG and IgM in transitional milk and IgA in colostrum, but not in mature milk (62). In our study of mature milk, IL-8 positively correlated with parity in the infected group. Chollet-Hinton et al. (2014) (63) reported higher cytokine levels in mature milk from multiparous women, but not IL-8. A negative correlation between lymphocyte and plasma cell counts and parity was also reported (64).

It has been discussed that primiparity may affect immune composition through birth-order effects linked to *in utero* immune programming (61, 65). It has been suggested that the reduced antibody concentrations in multiparous mothers were due to decreased B lymphocyte homing (60). However, this does not align with our findings. Although we observed an inverse correlation between plasma cells and parity, there was no correlation, either positive or negative, between IgA or IgG levels and parity. Nevertheless, the mechanisms underlying these associations still remain unclear and require further investigation.

In addition to their chemostatic role, chemokines also have antimicrobial functions *in vitro* and can kill human pathogens, including gram-negative and gram-positive bacteria, parasites, and fungi (66–69). The increase in several chemokines in milk during respiratory infection in nursing infants could provide an additional tool to address pathogens.

It is important to note that the role of milk leukocytes in breastfed infants remains unknown. It is well established, however, that human infant feces contain viable, functional breast milk leukocytes that have resisted gastric acid. It is suggested that in early infancy when the intestinal barrier is still permeable, milk leukocytes and immunoglobulins may cross this barrier, and migrate into infant lymphoid tissues, contributing to cell-mediated immunity in the lactation period (70).

It has been demonstrated in mice, lambs, and baboons, that milk leukocytes can pass into blood circulation by diapedesis (58, 71–74). Other studies also performed with different animal models have shown that milk maternal cells, including leukocytes, are found in the baby’s brain, spleen, mesenteric lymph nodes, liver, Peyer’s patches, thymus, pancreas, and blood (71–79). The implantation of live maternal cells into infant tissues during early lactation is an example of microchimerism and may be involved in an effective immune response, tissue repair, and regulation of the neonatal immune response and immune tolerance (80, 81). Additionally, it has been reported that Peyer’s patches exhibit greater permeability than other intestinal regions and can allow maternal milk cells to pass through (78). Evidence indicates that milk stem cells can cross the gut wall in nursing mice and rabbits, enter the bloodstream, and subsequently integrate into various organs where they become functional (82). The absence of MHC antigen expression on these maternal stem cells allows them to be easily accepted by infant tissues (82). Maternal stem cell microchimerism through breastfeeding in infants can produce Foxp3+ regulatory T cells (T reg), which inhibit antimaternal immunity (83). Indeed, those who were breastfed as newborns may accept maternal transplants more readily, as seen in hematopoietic stem cell transplantation (84).

A study on microchimerism through the placenta, which involves maternal-fetal cell exchanges, found that circulating fetal cells can persist in the mother for up to 27 years after delivery (85). Similarly, maternal peripheral blood mononuclear cells have been detected in offspring up to 62 years after birth (86). Furthermore, maternal milk cells have been observed in adult mice even after the full development of the intestine (77, 78). These studies demonstrate that microchimeric cells can survive for an extended period.

Although substantial evidence from animal models highlights the presence of maternal cells in offspring acquired during pregnancy and breastfeeding, much remains to be clarified regarding the impact and function of immune cells transferred through human breast milk on the health and development of nursing infants.

## 5 Conclusions

Our findings demonstrate that respiratory infections in nursing infants are associated with higher leukocyte counts, increased T and B lymphocyte numbers, and all their subtypes, along with elevated concentrations of chemokines, cytokines, IgA, and IgG in milk. This immunological boost in milk during infant infection could provide an additional strategy for passive maternal-infant protection against respiratory infections and may also support immunological tolerance and the maturation of the infant’s immune system.

## Data Availability

The original contributions presented in the study are included in the article/**Supplementary Material**. Further inquiries can be directed to the corresponding author.
