# Supplementary material for: Infant respiratory infections modulate lymphocyte homing to breast milk
Source: Front Immunol. 2025 Jan 10;15:1481416. doi: 10.3389/fimmu.2024.1481416 (PMC11757141; doi:10.3389/fimmu.2024.1481416)
Supplement: Supplementary file 1 [file Table1.docx]

**Supplement Table 1.** IL-1β, IL-10 and TNF-α levels in the serum and milk from mothers of infected and healthy (control) nursing infants.

|  | Serum | | | Milk | | | Infected | Control |
| --- | --- | --- | --- | --- | --- | --- | --- | --- |
| pg/mL | **Infected** | **Control** | ***p*** | **Infected** | **Control** | ***p*** | **B X M (*p*)** | **B X M (*p*)** |
| IL-1β | 0.2  (0-0.8) | 0.4 (0-0.6) | *0.7993* | 1.0  (0.6-1.9) | 1.1  (0.4-1.8) | *0.8325* | ***0.0058*** | *0.0679* |
| IL-10 | 0.3 (0-0.6) | 0.1 (0-0.4) | *0.3652* | 0  (0-0.4) | 0.2  (0-0.7) | *0.2069* | *0.2012* | *0.3273* |
| TNF-α | 0.5  (0.1-0.6) | 0.9  (0.1-1.2) | *0.0813* | 0.6  (0.3-1.0) | 0.6  (0.3-0.9) | *0.8958* | *0.1592* | *0.4758* |

B: Blood, M: Milk. Results expressed as medians (upper and lower CI 95%) analyzed by the Mann-Whitney Test. IL-12 was not detectable.

**Supplement Table 2.** Spearman correlation analysis to assess the relationship between the maternal age or parity and main results of milk from mothers of infected and healthy nursing infants.

|  | **Infected** | | | | **Control** | | | |
| --- | --- | --- | --- | --- | --- | --- | --- | --- |
|  | **Maternal  Age (r)** | ***p*** | **Parity (r)** | ***p*** | **Maternal  Age (r)** | ***p*** | **Parity (r)** | ***P*** |
| Maternal Age |  |  | 0.281 | *0.102* |  |  | -0.007 | *0.973* |
| Parity | 0.281 | *0.102* |  |  | -0.007 | *0.973* |  |  |
| IgA | -0.007 | *0.970* | 0.095 | *0.600* | 0.170 | *0.388* | 0.261 | *0.180* |
| IgG | 0.004 | *0.982* | -0.219 | *0.245* | 0.339 | *0.106* | 0.070 | *0.743* |
| CCL5 | -0.226 | *0.230* | 0.010 | *0.958* | 0.063 | *0.769* | 0.010 | *0.962* |
| CCL20 | -0.053 | *0.783* | 0.195 | *0.302* | 0.117 | *0.585* | -0.029 | *0.894* |
| CXCL10 | -0.054 | *0.782* | 0.170 | *0.377* | 0.148 | *0.501* | 0.217 | *0.319* |
| CCL28 | 0.100 | *0.591* | 0.018 | *0.925* | 0.104 | *0.620* | -0.264 | *0.203* |
| IL-8 | 0.193 | *0.273* | **0.382** | ***0.026*** | -0.119 | *0.538* | 0.072 | *0.712* |
| IL-6 | 0.040 | *0.823* | 0.035 | *0.845* | 0.192 | *0.319* | -0.089 | *0.647* |
| CD45 | -0.076 | *0.719* | -0.170 | *0.417* | 0.302 | *0.237* | -0.155 | *0.559* |
| T CD3 | -0.313 | *0.167* | -0.056 | *0.810* | -0.071 | *0.781* | 0.030 | *0.907* |
| T CD4 | -0.391 | *0.080* | -0.005 | *0.984* | -0.022 | *0.932* | 0.116 | *0.645* |
| T CD4 Naive | -0.372 | *0.097* | -0.061 | *0.792* | -0.171 | *0.496* | 0.377 | *0.123* |
| T CD4 Memory | -0.358 | *0.111* | 0.002 | *0.993* | -0.035 | *0.889* | -0.092 | *0.717* |
| T CD4 Activated | -0.302 | *0.196* | 0.053 | *0.823* | -0.241 | *0.349* | 0.113 | *0.668* |
| T CD8 | -0.287 | *0.221* | -0.193 | *0.414* | -0.050 | *0.844* | 0.022 | *0.930* |
| T CD8 Naive | -0.360 | *0.119* | -0.185 | *0.436* | -0.148 | *0.559* | 0.025 | *0.922* |
| T CD8 Memory | -0.149 | *0.543* | -0.122 | *0.617* | 0.125 | *0.622* | 0.025 | *0.922* |
| T CD8 Activated | -0.067 | *0.784* | -0.333 | *0.164* | -0.083 | *0.767* | 0.229 | *0.409* |
| B Lymphocyte | -0.124 | *0.539* | -0.129 | *0.520* | -0.161 | *0.509* | 0.116 | *0.638* |
| B Memory | -0.140 | *0.485* | -0.088 | *0.662* | -0.218 | *0.370* | 0.069 | *0.779* |
| B Naive | -0.009 | *0.964* | -0.070 | *0.727* | 0.070 | *0.777* | 0.286 | *0.235* |
| Plasma Cell | -0.304 | *0.234* | **-0.508** | ***0.039*** | -0.219 | *0.413* | -0.253 | *0.343* |

r = correlation coefficient. The significant p-values are in bold.
